# Adoptive cell therapy in combination with checkpoint inhibitors in ovarian cancer

**DOI:** 10.18632/oncotarget.27604

**Published:** 2020-06-02

**Authors:** Anders Handrup Kverneland, Magnus Pedersen, Marie Christine Wulff Westergaard, Morten Nielsen, Troels Holz Borch, Lars Rønn Olsen, Gitte Aasbjerg, Saskia J. Santegoets, Sjoerd H. van der Burg, Katy Milne, Brad H. Nelson, Özcan Met, Marco Donia, Inge Marie Svane

**Affiliations:** ^1^ National Center for Cancer Immune Therapy, Department of Oncology, Copenhagen University Hospital, Herlev, Denmark; ^2^ Section for Bioinformatics, DTU Health Technology, Technical University of Denmark, Kongens Lyngby, Denmark; ^3^ Center for Genomic Medicine, Copenhagen University Hospital, Copenhagen, Denmark; ^4^ Department of Medical Oncology, Oncode Institute, Leiden University Medical Center, Leiden, The Netherlands; ^5^ Deeley Research Centre, BC Cancer, Victoria, Canada; ^6^ Department of Medical Genetics, University of British Columbia, Vancouver, Canada

**Keywords:** adoptive cell therapy, tumor-infiltrating lymphocytes, ovarian cancer, combinational immune therapy, checkpoint inihibors

## Abstract

Immune therapy is a promising field within oncology but has been unsuccessful in ovarian cancer (OC). Still, there is rationale and evidence supporting immune therapy in OC. We investigated the potential for adoptive cell therapy (ACT) from in vitro expanded tumor-infiltrating lymphocytes (TILs) in combination with checkpoint inhibitors (ICI) and conducted immunological testing of *ex vivo* expanded TILs (REP-TILs).

Six patients with late-stage metastatic high-grade serous OC were treated with immune therapy consisting of ipilimumab followed by surgery to obtain TILs and infusion of REP-TILs, low-dose IL-2 and nivolumab.

One patient achieved a partial response and 5 others experienced disease stabilization for up to 12 months. Analysis of the REP-TILs with flow- and mass-cytometry show primarily activated and differentiated effector memory T cells. REP-TILs showed in vitro reactivity and expression of inhibitory receptors, such as LAG-3 and PD-1. Furthermore, our data indicate that addition of ipilimumab therapy improves the T cell fold expansion during production, increase the level of CD8 T cell tumor reactivity, and favorably affect the T cell phenotype.

We show that the combination of ICI and ACT is feasible and safe. With one partial response and one long-lasting SD, we demonstrated the potential of ACT in OC.

## INTRODUCTION

Ovarian cancers are frequently infiltrated with immune cells. T cell infiltration and, especially the number of CD8 T cells, is correlated to longer survival in ovarian cancer patients [1–3]. While ovarian cancer is characterized by a low to intermediate mutational burden [4, 5], a feature generally considered as an indication of low immunogenicity and responsiveness to immune therapy [6], we and others have demonstrated tumor reactivity amongst TILs [7] and peripheral blood lymphocytes [8] in ovarian cancer patients suggesting a potential for immune therapy.

Immune checkpoint inhibitors (ICI) has altered the landscape of cancer therapy in the recent decade, building a new independent pillar of immunotherapy. Several clinical trials have tested ICI in ovarian cancer patients but so far with very modest results. A study in 20 ovarian cancer patients showed an overall response rate (ORR) of 15% with the anti-PD-1 antibody nivolumab [9], but in a recently published, and much larger trial of 294 patients, the ORR was only 8% with the anti-PD-1 antibody pembrolizumab [10]. This is low when compared with the response rates of up to 37% in melanoma and 19% in NSCLC [11–13]. The anti-CTLA4 antibody ipilimumab has also been tested in ovarian cancer patients, claiming an ORR of 10.3% in a still unpublished trial (NCT01611558).

Adoptive cell therapy (ACT) using *ex vivo* expanded tumor-infiltrating lymphocytes (TILs) is an immune therapy modality that has been successfully pioneered within malignant melanoma in the 1980s and -90s. ACT has early on been tested in ovarian cancer patients with promising results in both the adjuvant [14] and metastatic setting [15]. Concomitantly, smaller phase I and II trials for other cancer diagnoses have confirmed clinical efficacy outside malignant melanoma [16, 17]. We recently published results from a small ACT pilot trial in ovarian cancer demonstrating feasibility but with no patients achieving objective responses [18]. Data from this trial indicated that the infused TILs had a high expression of the immune regulatory markers LAG-3 and PD-1.

The combination of different immune therapies is a natural next step and a promising field within oncology. An obvious and FDA approved example is the combination of nivolumab and ipilimumab that has shown a significantly increased clinical efficacy at the price of increased toxicity [19]. Ipilimumab is believed to prime and activate T cells early in the immune response [20] while the anti-PD-1 antibodies block PD-1 on already activated T cells which are directly inhibited by PD-L1 expression of tumor cells [21]. Mouse studies show that blockade of the CTLA-4 and PD-1 receptors synergistically induce CD4 and CD8 T cell numbers in the tumor microenvironment (TME) [22–24]. These findings indicate that checkpoint inhibition may be beneficial in the ACT setting and several clinical trials combining ACT with either CTLA-4 or PD-1 blockade are underway (NCT02027935, NCT03296137, NCT03638375, NCT03645928, NCT01993719). Here we report the clinical outcome of combining ACT with both CTLA-4 and PD-1 blockade in ovarian cancer patients.

## RESULTS

### Study population

Between 2016 and 2017, seven patients with late-stage and platinum-resistant high-grade serous ovarian cancer were recruited and underwent surgical tumor removal after ipilimumab infusion. One patient (#5) was discontinued shortly after surgery due to rapid cancer disease progression and associated clinical deterioration. Six patients were treated with *ex vivo* expanded T cells (REP-TILs). The baseline patient characteristics are listed in Table 1.

**Table 1:** Baseline patient characteristics

ID	Age	Histology	Sites of disease	Prior systemic treatment	Prior lines
1	51	Serous adenocarcinoma	Peritoneum, vagina, lymph nodes	Carboplatin/paclitaxel, doxorubicin, paclitaxel/bevacizumab, tisotumab vedotin	4
2	52	Serous adenocarcinoma	Peritoneum, lymph nodes	Carboplatin/paclitaxel, carboplatin/doxorubicin/bevacizumab, carboplatin/gemcitabin, topotecan	4
3	63	Serous adenocarcinoma	Peritoneum, lung, pleura, lymph nodes,	Carboplatin/paclitaxel, carboplatin/paclitaxel/bevacizumab, doxorubicin, cabazitaxel, topotecan	4
4	60	Serous adenocarcinoma	Peritoneum, lymph nodes	Carboplatin/paclitaxel, carboplatin/paclitaxel/bevacizumab, doxorubicin, paclitaxel, gemcitabin, trabectidin, tisotumab vedotin	7
6	49	Serous adenocarcinoma	Peritoneum, lung, liver, lymph nodes	carboplatin/paclitaxel/bevacizumab, doxorubicin, paclitaxel, topotecan	4
7	54	Serous adenocarcinoma	Peritoneum, lymph nodes	Carboplatin/paclitaxel, bevacizumab/caelyx, trabectidin	3

### Feasibility and safety

All patients received ipilimumab prior to tumor removal. Tumor harvest and *ex vivo* expansion of TILs were successful in all patients. One patient (#6) could not undergo surgery and instead a double liver biopsy (2 mm) was performed. Four patients underwent laparoscopic surgery to resect intraperitoneal metastases, and one patient had a lung metastasis removed. The median expansion time before rapid expansion protocol (REP) was 25 days (Range: 18–42 days). The therapy and expansion data are listed in Table 2.

**Table 2:** Summary of expanded TILs (REP TILs), therapy and clinical response

ID	Metastasis location	Exp. time (days)	Surgery to ACT (days)	Infused cells (10e9)	Fold exp.	CD3 (%)	CD4 (%)	CD8 (%)	IL-2 doses	Nivo-lumab doses	BOR	PFS (days)	OS (days)
1	Vagina	18	34	107	5360	99.7	26.4	70.2	14	4	PR	99	685
2	Peritoneum	42	65	21.9	1441	98.6	45.8	42.5	14	4	SD	342	489
3	Both ovaries	21	58	93.9	4680	99.5	36.1	58.0	4	1	SD	86	214
4	Peritoneum	25	62	75.0	3790	99.5	96.2	3.1	11	3	SD	84	280
6	Liver	27	50	98.4	4920	93.6	7.8	88.5	14	2	SD	144	144
7	Peritoneum	28	93	54.4	2720	99.1	73.0	20.9	14	4	SD	86	136
Median		25	60	84.7	4235	99.3	41.0	50.2	14	3.5		86	214

Four patients received all Interleukin-2 (IL-2) doses (median: 14; range: 4–14) following the T cells and 3 patients received all 4 doses of nivolumab (median: 3.5; range: 1–4). In two patients, IL-2 therapy was prematurely discontinued due to respiratory toxicity (#3) and dizziness (#4). In three patients, nivolumab was prematurely discontinued due to patient’s wish (#4) and general deterioration in clinical condition (#3 and #6). The median admission time was 20.5 days (Range: 16–31 days).

Overall the treatment was tolerated with manageable and expected toxicities associated with T cell therapy including conditioning chemotherapy [25–28]. All grade 3-4 adverse events (AEs) are listed in Table 3. Most adverse events occurred during hospitalization for chemotherapy and T cell infusion. The median length of neutropenia was 5.5 days (range: 5–9 days) and all patients required either blood- or platelet transfusions. The infusion of autologous T cells was well tolerated. The most common adverse events were fever, chills and dyspnea. One patient (#2) experienced severe respiratory deficiency managed at the intensive care unit. This patient had severe bilateral pleuritic fluid before T cell infusion that was deemed undrainable due to fluid compartmentalization.

**Table 3:** Safety and toxicity. List of grade 3 and 4 adverse events related to study drugs (CTCAE 4.0)

Therapy	Adverse event (Grade 3-4)	*n*
*Chemotherapy*	Performance status 3-4	3
	Fatigue	3
	Nausea	1
	Vomiting	1
	Diarrhea	1
	Hyponatremia	3
	Infection	2
	Neutropenia	6
	Anemia	6
	Trombocytopenia	6
	Agammaglobulimia	1
*T cells (REP-TILs)*	Dyspnea	1
*IL-2*	Performance status 3-4	3
	Fever	3
	Fatigue	2
	Vomiting	1
*Checkpoint inhibitors*	Colitis	1
	Thyroiditis (grade 2)	1
	Dry skin	1

The low-dose IL-2 regime was also generally well tolerated. The primary adverse events were fever, fatigue, dyspnea, nausea and vomiting. Two patients experienced immune-related adverse events (IrAEs) following the ICI treatment, which are attributed to these drugs. One patient (#7) developed grade 3 colitis following the single dose ipilimumab, and T cell therapy had to be postponed until completion of prednisolone therapy.

### Clinical efficacy

**Figure 1:**
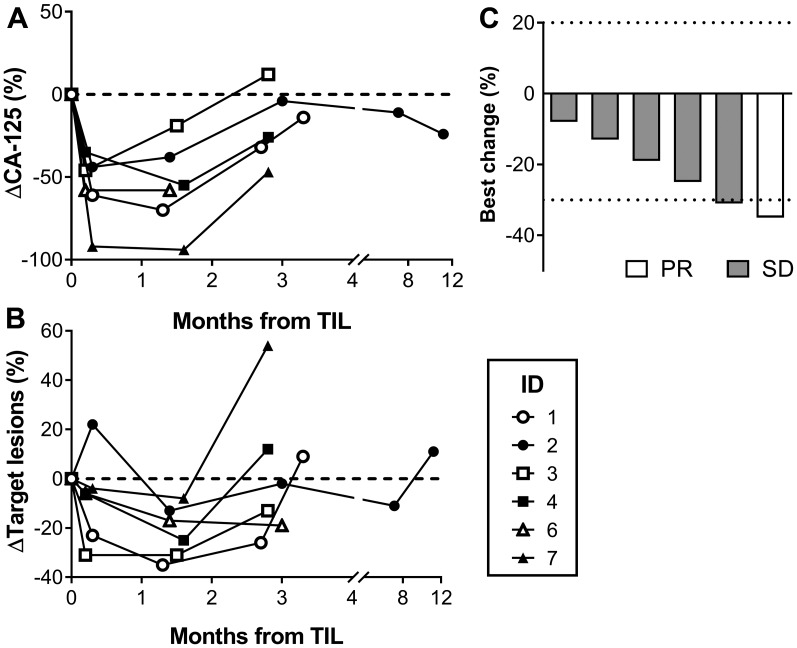
Clinical response. Clinical response curves following the infusion of *ex vivo* expanded TILs. (**A**) shows the proportional change of the cancer antigen-125 (CA-125) (**B**) shows radiological change in the target lesion sum according to RECIST 1.1, and (**C**) is a waterfall plot with the best overall response (BOR).

The best overall tumor response (BOR) comprised 1 partial response (PR) in patient #1 and stable disease (SD) in the remaining 5 patients, whereof patient #3 had an unconfirmed PR. Tumor regression was achieved in all patients (8-35% size decrease in target lesions) early after the T cell infusion with a median PFS of 86 days. At 12 weeks, 4 out of 6 patients had progressive disease (PD). One patient (#2) had long-lasting SD for almost a year following the therapy. In general, serum levels of CA-125 correlated with tumor response on imaging. The clinical efficacy is shown in Figure 1.

### Phenotypic characterization of expanded TILs

The phenotype of REP-TILs was characterized with flow- and mass cytometry. The infused cells were almost exclusively T cells with a median of 99.3% (range: 93.6–99.7%) of live cells. In 3 patients CD8 T cells were the dominant subtype, including the two patients with objective responses, while CD4 T cells were dominant in the others, including the patient with long-lasting SD as listed in Table 2.

**Figure 2:**
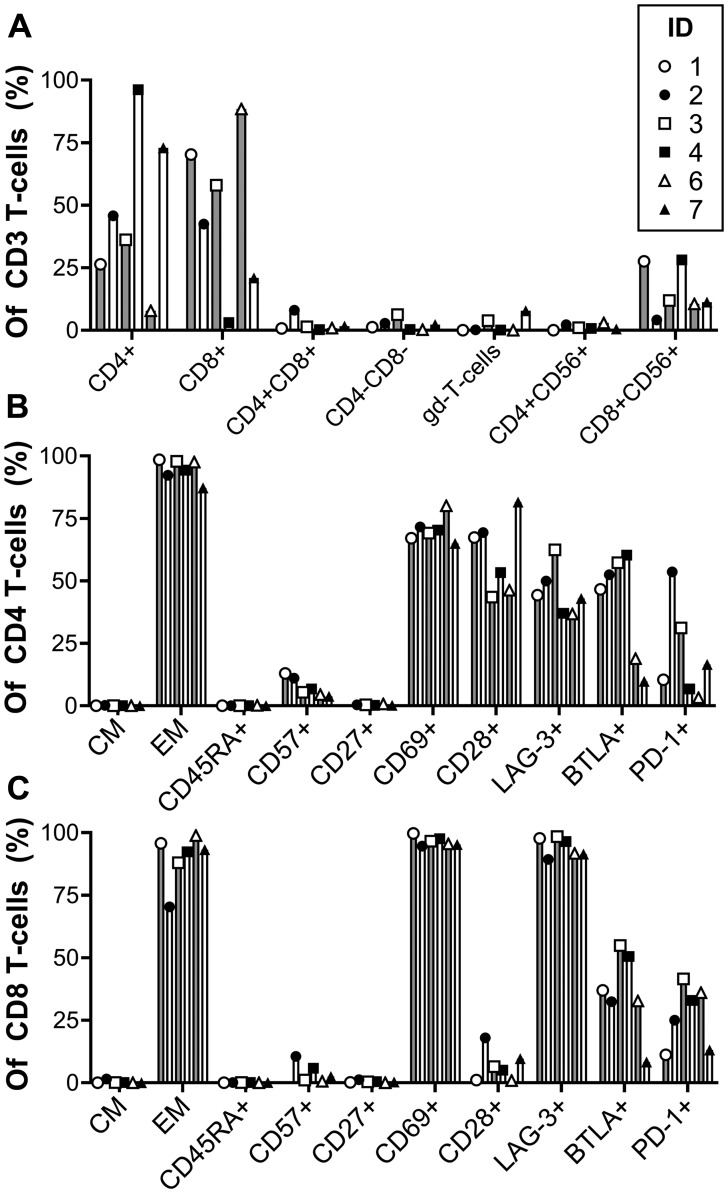
Phenotype of *ex vivo* expanded tumor infiltrating lymphocytes (REP-TILs). The combined flow cytometry results showing the different cell populations within the CD3+ T cells (**A**), CD4+ T cells (**B**) and CD8+ T cells (**C**). Gd: Gamma-delta; CM: Central memory (CD45RA-CCR7+); EM: Effector memory (CD45RA-CCR7-).

The REP TILs were almost exclusively effector memory (EM; CD45RA-CCR7-) T cells and had an overall negligible CD45RA expression. Both CD4 and CD8 T cell subsets were primarily CD27-, CD28- and CD69+ which generally are considered marks of activation and differentiation. In contrast, the CD28 expression on CD4 T cells (median of 60.3%) together with a low CD57 expression (CD4 median: 6,1%; CD8 median: 1.8%) indicated less differentiation. The expression of the immune regulatory checkpoints BTLA, PD-1, and especially LAG-3, was high, respectively 49.5%, 13.5% and 43.6% in CD4 T cells and 34.8%, 29% and 94.1% in CD8. Thus, the CD8 T cells were generally more activated and differentiated than the CD4 T cells and expressed more LAG-3 and PD-1. All results are shown in Figure 2.

**Figure 3:**
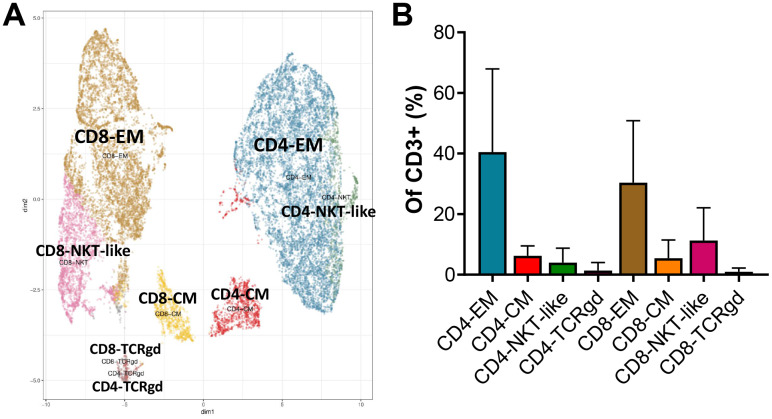
Mass cytometry of *ex vivo* expanded tumor infiltrating lymphocytes (REP-TILs). Semi-supervised clustering analysis based on CD4, CD8, CD45RO, CCR7, CD45RA, CD56, HLA-DR and TCRgd expression as lineage markers. (**A**) shows the Umap of the 8 resulting clusters while (**B**) shows the proportional size (median with range) of the clusters.

Mass cytometry and clustering analyses were performed on REP-TILs from this and another clinical pilot trial, in which no ipilimumab treatment before surgery was used, resulting in *n =* 12 samples, which allowed better clustering of T cell subsets. As shown in Figure 3 and Supplementary Table 3, we found 8 T cell clusters based on the expression of the eight lineage markers, all of which expressed HLA-DR. The infused T cells were primarily CD4 and CD8 EM T cells with medians of 37.1% (Range: 6.3–82.5%) and 29.1% (Range: 3.9–62.3%) of CD3+ cells respectively.

In contrast to our FACS data, we also found a considerable number of central memory (CM; CD45RA-, CCR7+) T cells with a median (of CD3+) of 7.4% (range: 0.9–9.9%) in CD4 T cells and 3% (range: 0.4–19.6%) in CD8. The other clusters were CD56+ T cells (NKT-like cells), median of 2.5% for CD4 (range: 0.6–18.3%) and 8% for CD8 (range: 0.7–35.5%), and a negligible number (medians ≤ 0.5%) of gamma-delta T cells (TCRgd cells). All cells clustered expressed HLA-DR as a sign of profound activation. The Uniform Manifold Approximation and Projection (UMAP) and the clusters are shown in Figure 3 and listed in Supplementary Table 3, while the expression of non-lineage markers, heatmap and density plots are shown in Supplementary Figure 1 and 2.

### Tumor expression of selected immune markers

**Figure 4:**
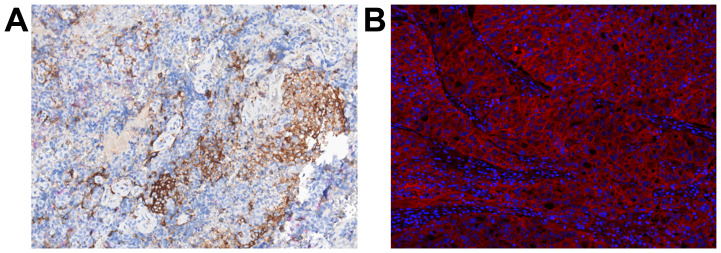
Immunohistochemistry and immunofluorescence of tumor tissue. (**A**) shows the PD-1/PD-L1/CD163/Hematoxylin staining of the tumor tissue from patient #1, where PD-L1 is shown in brown, CD163 in blue, PD-1 in red and hematoxylin nuclear counterstain in dark blue. (**B**) is a component fluorescence image generated by inForm spectral unmixing, from the MHCI/MHCII/IDO/DAPI staining, showing the MHC II class expression (in red) in the tumor tissue from patient #3 and nuclear counterstain DAPI in blue.

Next, we investigated the tumor expression of, among others, the immune markers PD-L1, MHC class I and II. In 5 patients surplus tissue from the tumor resection was fixed and available for IHC and IF analysis. We found a high MHC I and II expression (>30 average count) in patient #1, #2 and #3. Tumor expression of PD-L1 (CD163-PD-L1+; >25 average count) was found in patient #1, #2, #3 and #4. Examples of the IHC and IF analysis are shown in Figure 4. Interestingly, MHC class I and II expression were highly expressed in the 3 patients that achieved objective response, unconfirmed PR or sustained SD. The tumor expression of PD-L1, MHC class I and II was not significantly different from what was seen in our prior pilot study of patients without treatment with ipilimumab (data not shown).

### TIL anti-tumor reactivity

**Figure 5:**
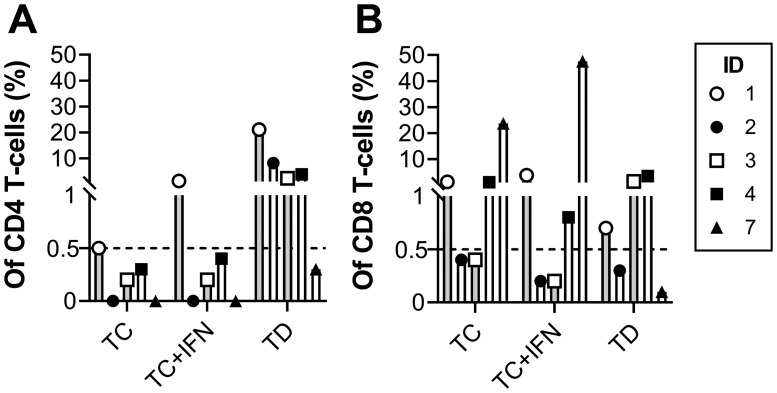
***In vitro*** **tumor reactivity of expanded TILs.** Proportional number of reactive CD4 (**A**) and CD8 (**B**) T cells after 4 hours of co-culture with either cultured tumor cells (TC), cultured tumor cells with IFN-gamma (TC+IFN) or tumor digest (TD). Tumor reactivity was defined as the production of TNF and/or IFN-gamma and/or expression of CD107 as assessed by flow cytometry with >0.5% tumor reactive T cells and >50 events.

The *in vitro* tumor reactivity of the REP TILs was tested using autologous tumor cells. Tumor cells were either tumor cell culture (TC) or tumor digest (TD) established from the resected tumor tissue used for TIL expansion. Tumor samples were available from 5 patients. All patients had either CD4 or CD8 tumor-reactive T cells. As shown in Figure 5, the highest reactivities were seen within the CD8 T cells, and the CD4 T cells were almost exclusively reactive against TD. Patient #1, #2, #3 and #4 showed CD4 anti-tumor reactivity, with respectively 21.1%, 8.2%, 2.3% and 3.8% positive cells, against TD. Patient #1 and #4 showed CD8 reactivity against TD and TC, patient #3 only against TC treated with interferon-gamma (IFN), while patient #7 showed extensive reactivity against TC and TC-IFN with respectively 23.7% and 47.6% anti-tumor reactive CD8 cells.

### Influence of ipilimumab treatment on TIL expansion and function

We compared the REP-TILs from the current patients with those from our previous pilot trial without ipilimumab treatment [18] to assess the influence of ipilimumab. The success rate of *ex vivo* T cell expansion was comparable and there was no significant difference in expansion time until REP. Though not significant, the median fold expansion was 4,235 in ipilimumab treated patients compared to 2,810 in the untreated leading to a higher number of infused T cells (median 84.7 × 10e9 cells compared to 56.2 × 10e9 cells) in ipilimumab treated patients. In the ipilimumab treated patients, the median CD8 T cell content in the final TIL product was 50.2% vs. 25% (n. s.) in untreated patients and a CD4 T cell content of 41% vs. 64.8% (n. s.) respectively, resulting in median CD8: CD4 ratios of 1.3 vs. 0.5 (n. s.). The immune phenotype was slightly altered in ipilimumab treated patients with lower LAG-3 (*p =* 0.002) in the CD4 T cells and lower CD27 (*p =* 0.002), CD28 (*p =* 0.015) and BTLA (*p =* 0.015) expression in the CD8 T cells. The phenotype comparison is shown in Supplementary Figure 3. The mass cytometry did not show significant differences between the clusters of ipilimumab treated and not treated patients. The sizes of the different clusters are listed in Supplementary Table 3. Antitumor reactivity of REP-TILs was found in 6 out of 6 of the ipilimumab treated patients compared to 4 out of 6 in patients not treated with ipilimumab and with a generally higher level of CD8 reactivity in ipilimumab treated patients (*p =* 0.048) with a pooled median of 0.8% (range: 0.1–47.6%) vs. 0.3% (range 0.1–1.6%).

## DISCUSSION

This study was established to examine the feasibility and potential benefit of adding ICI at specific time points during TIL ACT in ovarian cancer. We demonstrate that the investigated combination of checkpoint inhibition and ACT is a safe and feasible approach for immune therapy. Importantly, indication of clinical activity was seen with regression of target tumor lesions in all patients and two patients achieving a partial response (one unconfirmed). In addition, one patient achieved prolonged disease stabilization for almost a year. In the remaining patients the tumor responses were short-lived. It cannot be ruled out that clinical responses were partly or mainly induced by the supportive therapy with chemotherapy, ICI and/or low dose IL-2. However, in our previous ACT trial in ovarian cancer with the same chemotherapy schedule, and high dose IL-2 (decrescendo) but without checkpoint inhibition, no patients achieved objective response despite being less heavily pretreated [18]. Thus, even though short-lived responses could be associated with chemotherapy, we would not expect the chemotherapy to elicit significant anti-neoplastic effects in these late-stage ovarian cancer patients who all have progressed on several previous lines of chemotherapy. In this situation, it is also reasonable to assume that their disease would be very difficult to treat even with another, and otherwise promising, new therapy.

The treatment-related toxicity was manageable and largely comparable with our previous ACT studies besides the emergence of immune-related adverse events in two of the patients. These are anticipated with the use of checkpoint inhibitors [19]. Regarding lymphodepletion after preconditioning ipilimumab no specific disadvantages were observed. Thus, all patients reconstituted their immune system with a median time of neutropenia of 5.5 days which is less than melanoma patients without pretreatment with ipilimumab but with high dosis IL-2 [28, 29], and less than our previous trial in ovarian cancer patients, also with high dose IL-2, where the median time of neutropenia was 9 days (range: 8–10) [18]. One patient acquired a prolonged secondary B-cell deficiency (agammaglobulimia) that was attributed to the lymphodepleting chemotherapy [30].

Only half of the REP-TILs were CD8 dominated, and both patients with objective responses were treated with CD8 dominated REP-TILs. CD8 T cells are generally associated with anti-tumor immunity, and earlier data in melanoma indicate that the CD8 numbers correlate to better responses in ACT [31]. However, as our *in vitro* data show, both CD4 and CD8 can mediate anti-tumor-reactivity, which is also in line with earlier findings [17, 32, 33]. To this end, the patient with PR after therapy had anti-tumor reactivity in 21.1% CD4 REP-TILs and the patient with prolonged SD for almost a year was treated with a CD4 dominated TIL product with anti-tumor reactivity in 8.2% of CD4 REP-TILs, while both showed modest CD8 anti-tumor reactivity.

The immune phenotyping of the REP-TILs showed that they were primarily effector memory (EM) T cells with an activated and differentiated phenotype. There were some exceptions such as the high CD28 expression on CD4 T cells and the general lack of CD57. In ACT of melanoma patients, data from our center (unpublished) and others have shown that high CD28 expression is a positive predictor of *in vivo* activity in TIL-based ACT [25]. The appearance of a CM T cell subset in the mass cytometry data is most likely due to the difference in method, including a different anti-CCR7 clone, and automated clustering analysis.

Similar to our previous study [18], we found a considerable expression of the immune checkpoints LAG-3, BTLA and PD-1. The PD-1 expression led us to add an anti-PD1 antibody to the ACT in the current trial. LAG-3 and BTLA are expressed on activated lymphocytes [21]. LAG-3 works primarily through binding of its ligand MHC class II [34] and is, together with PD-1, an important regulator of TILs in ovarian cancer [35]. MHC class I and II expression were found in several patients and coincided with favorable clinical responses. On the other hand, the co-expression of MHC class II on tumor cells and LAG-3 on CD8 REP-TILs is a potential limitation for the anti-tumor reactivity of REP-TILs and suggests that the addition of an anti-LAG-3 blocking antibody might be beneficial in these patients. Similarly, the importance of BTLA in the microenvironment of ovarian cancer has recently been reported [36] and, though the immune regulatory pathway might be more complex, anti-BTLA therapy might also be beneficial in ACT [37]. Still, two patients showed no tumor expression of MHC class I or II which could limit the tumor recognition and T cell reactivity altogether.

Patient preconditioning with the anti-CTLA-4 antibody ipilimumab was employed to increase tumor infiltration of tumor reactive lymphocytes before tumor tissue harvest for TIL production. To address the potentially beneficial effect of ipilimumab we compared TIL data from the present pilot study with data from our previous ovarian cancer pilot study without ipilimumab. Due to the low number of patients and the fact that they were separate clinical trials, we were not able to draw firm conclusions, however, data indicated several important potential beneficial effects including increased success rate of *ex vivo* TIL expansion and an improved quality of the TIL product comprising increased total TIL numbers, a more favorable CD8: CD4 ratio, and increased antitumor reactivity. Other studies indicate a similar influence of ipilimumab therapy on TILs [38, 39](Friese *et al*., Scientific Reports, in press). On the other hand, our data show that ipilimumab treatment seems to lower CD27 and CD28 expression on CD8 T cells, while decreasing BTLA expression on CD8 T cells and LAG-3 on CD4 T cells, which indicates that ipilimumab results in REP TILs that are more differentiated, but with lower expression of immunological checkpoints. A comprehensive study of differences in TME dependent on pre-conditioning ipilimumab in these patients, including multiplex and nanostring analyses are subject of a separate study (Westergaard et al. Manuscript in preparation)

ACT for ovarian cancer is still in its cradle compared to malignant melanoma that is presently in phase III clinical testing (NCT02278887). However, accumulative evidence points towards a potential role of immunotherapy in ovarian cancer but with a modest efficacy of established ICIs. Thus, combination immunotherapy might be a way forward supporting a continuous development and refinement of ACT therapy for this purpose.

## MATERIALS AND METHODS

### Study design

**Figure 6:**
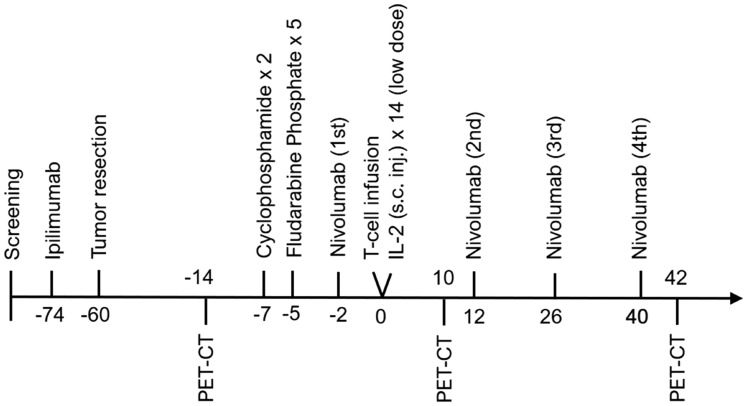
Therapy regimen. Overview and timeline of the trial with conditioning chemotherapy, adoptive cell therapy and checkpoint inhibitors during the clinical trial.

TIL therapy was combined with checkpoint inhibition in a sequenced manner. The patients received one dose of ipilimumab (3 mg/kg) 2 weeks prior to tumor resection for *ex vivo* TIL expansion. When the expanded cells reached sufficient numbers (>20 × 10e6 cells), they were transferred to a rapid expansion protocol (REP) and the patients were admitted for therapy with conditioning chemotherapy, T cell infusion, nivolumab (3 mg/kg; q2w × 4) and low-dose IL-2 (2 MIE s. c. daily for 2 weeks). The conditioning chemotherapy consisted of 2 days with cyclophosphamide (60 mg/kg) followed by 5 days with fludarabine phosphate (25 mg/m^2). The study design is illustrated in Figure 6. The patients were monitored daily and scored for adverse events (CTCAE v4.0) during hospitalization and before every new therapy.

The primary endpoints were safety and feasibility of the therapy regimen. Secondary endpoints were radiologic response evaluation (RECIST 1.1) and immune monitoring. Radiologic imaging was performed at approximately 2, 6 and 12 weeks following T cell infusion, thereafter every 3 months.

### Patients

Patients were recruited and treated at Herlev Hospital, Copenhagen, Denmark. Eligible patients must have exhausted available standard therapy options and have tumor lesions available for both surgical removal and response evaluation. Other inclusion criteria include: Pathologic diagnosis with epithelial ovarian cancer, ECOG performance status of 0–1, permissible organ function, absence of chronic infections and absence of brain metastases.

Oral and written informed consent was obtained from all patients in accordance with the declaration of Helsinki. The study was approved by the National Ethics Committee, the Danish Medicines Agency and the Danish Data Protection Agency before commencement. The clinical trial is registered at clinicaltrials. gov (NCT03287674) and EudraCT (2017-002179-24).

In a previous clinical trial, we treated 6 ovarian cancer patients with TIL therapy without checkpoint inhibitors [18]. Data and material from this study were included for comparative analyses to e. g. investigate the effect of the ipilimumab treatment on the expanded T cells.

### *Ex vivo* TIL expansion

Resected tumor lesions were immediately transported from the department of surgery to a GMP facility at Herlev Hospital for further processing. The tumors were manually dissected into 48 fragments and cultured in two 24-well plates (Nunc, Roskilde, Denmark) with complete medium (CM) containing; RPMI (Invitrogen, Waltham, MA), 1% penicillin and streptomycin, 0.5% Fungizone (Bristol-Myers Squibb, New York, NY), 10% Human serum (Sigma-Aldrich, St. Louis, MO) and 6000 U/mL IL-2 (*Aldesleukin;* Novartis, Basel, Switzerland). The CM was exchanged every 2–3 days until reaching approximately 50 × 10e6 cells, when the cells were either cryopreserved for later use or further expanded in a rapid expansion protocol (REP). In the REP 20 × 10e6 cells, together with irradiated (40 Gy) allogeneic feeder cells at a 1:200 ratio, were cultured in 80/20 medium (80% CM and 20% AIM-V medium) supplemented with 30 ng/mL anti-CD3 antibody (*OKT3*; Miltenyi Biotech, Bergisch Gladbach, Germany) and IL-2 at a concentration of 6000 U/mL. After 7 days, the cells were moved from a static expansion to the dynamic Wave^®^ system (GE Healthcare, Chicago, IL). After another 7 days of culture, the cells (REP-TILs) were infused into the patient. The method is further elaborated elsewhere [40] and identical to our previous clinical trial in ovarian cancer.

### Immune characterization of expanded TIL with flow- and mass cytometry

For flow cytometry, REP-TILs were thawed and rested overnight in CM. Approximately 15 × 10e6 cells were washed in phosphate-buffered saline and stained at 4° C for 30 minutes. The fluorochrome-labeled monoclonal antibodies and panels are listed in Supplementary Table 1. The stained cells were analyzed on a FACS Canto II instrument (BD Biosciences).

Mass cytometry was performed at LUMC (Leiden, Netherlands). Approximately 2.5 × 10e6 REP-TILs were stained with metal-labeled antibodies as described elsewhere [41]. The antibody panel is listed in Supplementary Table 2. Cells were first stained for 15 min with a live/dead identifier 1 mmol/L Cell-ID intercalator-103Rh (Fluidigm, San Francisco, CA) and washed with MaxPar Cell stain buffer (Fluidigm). The cells were incubated with Human TruStain FcX Fc Receptor Blocking Solution (Biolegend, San Diego, CA) for at least 10 min before staining with the metal-conjugated antibodies and incubated for 45 min, followed by two washes with the MaxPar Cell stain buffer. The cells were stained overnight at 4° C with 125 nmol/L Cell-ID intercalator-Ir in MaxPar Fix and Perm Buffer (Fluidigm). The following day, the cells were washed and acquired by CyTOF 2 or helios-upgraded CyTOF 2 mass cytometer (Fluidigm). Data were then normalized by using EQ Four Element Calibration Beads (Fluidigm) with the reference EQ passport P13H2302.

Available REP TIL samples from our previous clinical trial in ovarian cancer patients [18] were analyzed with mass cytometry together while the flow cytometry was performed at different time points but with near-identical antibody panels.

### Tumor immunohistochemistry and immunofluorescence

Formalin-fixed paraffin-embedded (FFPE) tumor tissue samples were analyzed with multicolor IHC and immunofluorescence (IF) analysis. Methods and reagents are described in our previous TIL study [18]. All reagents were obtained from Biocare Medical (Pacheco, CA) unless otherwise listed. Slides were first deparaffinized through xylene and graded alcohols to water then subjected to antigen retrieval with Diva Decloaker.

HLA-I/HLA-II expression was analyzed with multicolor IF using OPAL reagents (Perkin Elmer, Waltham, MA). The manufacturer’s instructions were followed. Briefly, HLA Class I A, B, C (clone EMR8-5, MBL, Woburn, MA) on OPAL520 was used in round 1, HLA-DP, DQ, DR (clone CR3/43, Affinity Bioreagents, Golden, CO) on OPAL650 in round 2 and IDO-1 (clone SP260, Spring Bioscience, San Francisco, CA) on OPAL570 in round 3. The staining with primary antibody was followed by staining with suitable MACH2 polymers (Biocare Medical).

To assess PD-L1/CD163 expression, a cocktail of anti-PD-L1 (clone SP142, Spring Bioscience, Pleasanton, CA) and anti-CD163 (clone 10D6, Biocare) in Da Vinci Green diluent was added for 30 minutes followed by MACH2 Double Stain # 1 Polymer for 30 minutes. Before staining, Peroxidased-1 and then Background Sniper were added to block non-specific staining. The signal was detected by adding IP Ferangi blue followed by IP DAB. Slides were then subjected to denaturation at 50° C for 45 minutes in an SDS-glycine pH2.0 solution. Slides were then incubated with anti-PD1 (clone EPR4877 [2], Abcam) in Da Vinci Green diluent for 30 minutes followed by MACH2 Rabbit-AP Polymer for 30 minutes then IP Warp Red Chromogen. Slides were then counterstained with a 1:5 dilution of CAT hematoxylin, rinsed, air-dried and coverslipped with Ecomount.

### *In vitro* tumor reactivity

When possible, tumor cell lines were established from the remaining transport medium after tumor fragmentation. The tumor cells were expanded in RPMI (Invitrogen), 1% penicillin and streptomycin, 0.5% Fungizone (Bristol-Myers Squibb), 10% FBS (Gibco, Nærum, Denmark). Some cell lines were additionally supplemented with insulin and sodium pyruvate. A tumor digest sample was prepared from tumor fragments that were enzymatically digested overnight using collagenase type IV (Sigma-Aldrich) and Pulmozyme (Roche, Basel, Switzerland) followed by cryopreservation.

REP TILs were tested for their ability to recognize either tumor digest or autologous cultured tumor cells with or without IFN-gamma stimulation. All antibodies were from BD bioscience unless otherwise listed. Briefly, TILs and tumor cells were co-cultured (3:1) for 5–7 hours in the presence of Golgi plug (BD biosciences) and CD107a-BV421. Cells were washed twice and stained with extracellular antibodies CD3-FITC, CD4-PerCP (Biolegend), CD8-Qdot605 (Invitrogen), CD56-PE, and Near-IR live-dead (Thermo Fisher, Waltham, MA) for 40 min followed by a wash. Fixation and permeabilization buffer (eBioscience, San Diego, CA) were added for overnight incubation at 4°C. The next day, cells were washed in fixation buffer and stained with intracellular antibodies TNF-APC and IFN-gamma-PE-Cy7 for 40 min. The cells were acquired on a FACS Canto II (BD biosciences). The method is described in more detail elsewhere [7].

### Data and statistical analysis

T cell subsets from the FACS data were gated using Flowjo software (BD biosciences). Differential abundance of subsets was tested with Mann–Whitney *U* test and *p*-values < 0.05 were considered statistically significant.

The mass cytometry data was manually gated for single, live and CD45+ cells using the cloud-based Cytobank software (Fluidigm) and exported as FCS files. A multidimensional unsupervised clustering analysis [42] was done using the algorithm flowSOM [43] for R (R Foundation for Statistical Computing, Vienna, Austria) using CD4, CD8, CD45RA, CCR7, CD45RA, CD56, HLA-DR and TCRgd as lineage markers. Dimensionality reduction visualizations were generated using Uniform Manifold Approximation and Projection (UMAP) [44]. Statistical analysis of differential abundance of cell subsets was done with Negative Binomial Generalized Linear Model and *p*-values > 0.05 after multiple testing correction using Benjamini Hochberg false discovery rate were considered statistically significant.

In the tumor reactivity analysis, tumor reactivity was defined as the production of TNF-α and/or IFN-gamma and/or expression of CD107a. REP-TILs without tumor stimulation were used as a negative control and subtracted from the tumor reactivity results. A positive anti-tumor response was defined as >0.5% positive of CD4 or CD8 T cells with >50 events.

Vectra 2 Multispectral Imaging system (Perkin Elmer, Waltham, MA) was used for imaging of slides. Ten 20× images per slide that had at least 90% tissue content in the field of view were collected. InForm image analysis software (Perkin Elmer) was used for the cell phenotype analysis. For each panel, 5 algorithms were developed, and the results were visually validated to ensure accuracy. Significant differences were tested with Mann-Whitney *U* test and *p*-values >0.05 were considered statistically significant.

## CONCLUSIONS

In this pilot study, we demonstrate the anti-tumor reactivity potential of unselected REP-TILs in ovarian cancer patients. Our findings suggest that checkpoint inhibition is advantageous in this ACT setting in terms of TIL production and clinical efficacy.

## SUPPLEMENTARY MATERIALS


