# Supplementary material for: Adoptive cell therapy in combination with checkpoint inhibitors in ovarian cancer
Source: Oncotarget. 2020 Jun 2;11(22):2092–105. doi: 10.18632/oncotarget.27604 (PMC7275789; doi:10.18632/oncotarget.27604)
Supplement: Supplementary file 1 [file oncotarget-11-2092-s001.pdf]

Adoptive cell therapy in combination with checkpoint inhibitors in ovarian cancer

SUPPLEMENTARY MATERIALS

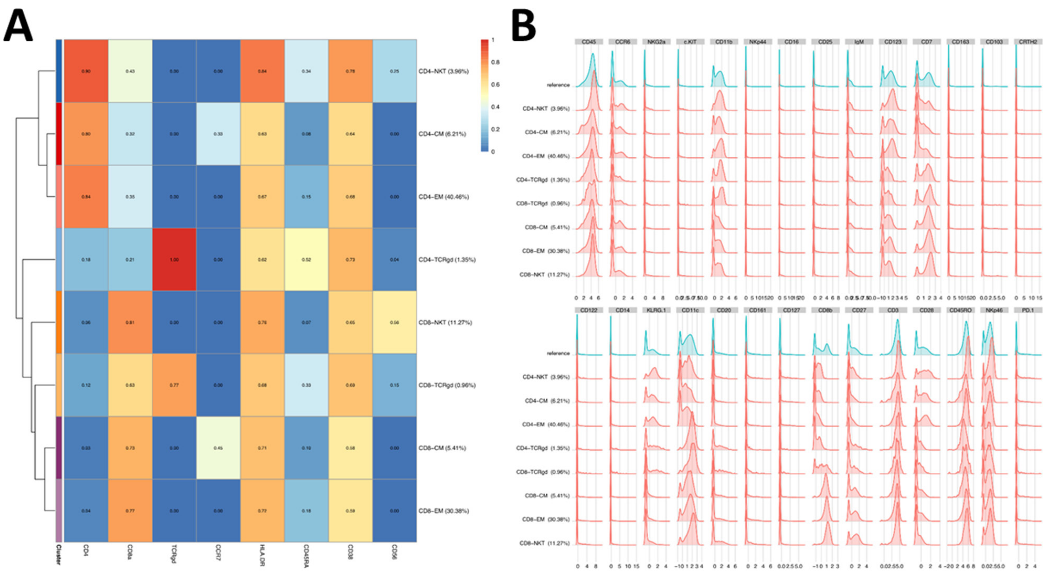

Supplementary Figure 1: Heatmap (**A**) and density plots (**B**) of the mass cytometry data. The expression of the individual lineage markers in the 8 clusters of the *ex vivo* expanded tumor infiltrating lymphocytes (REP-TILs) after cluster analysis.

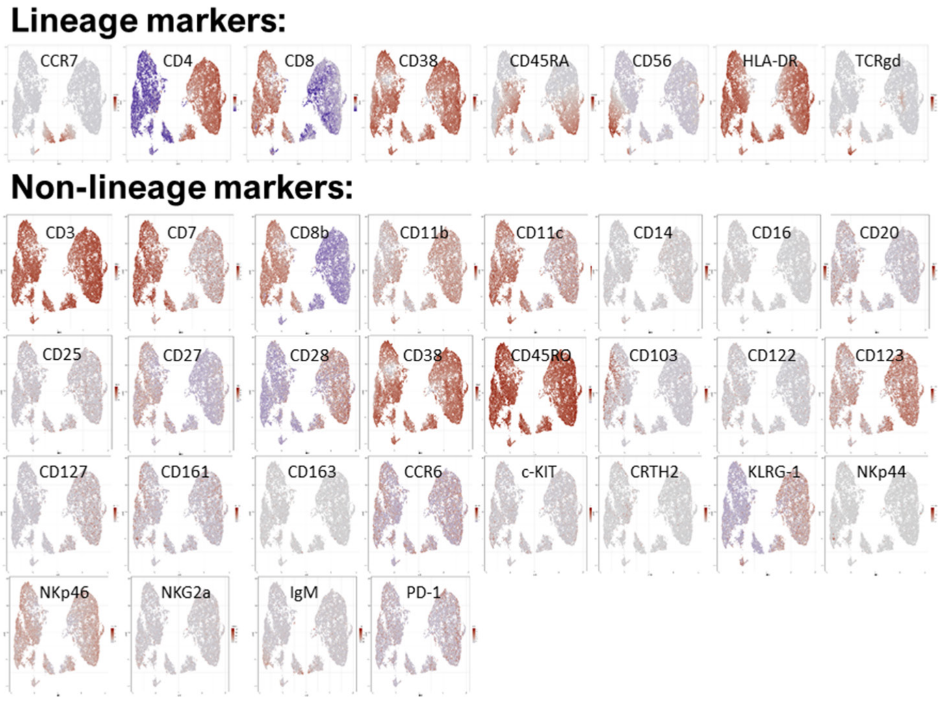

Supplementary Figure 2: Lineage and non-lineage markers from the mass cytometry. Pooled expression of lineage and non-lineage markers of the REP TILs after cluster analysis. The red color indicates high expression of the individual marker while blue is low expression.

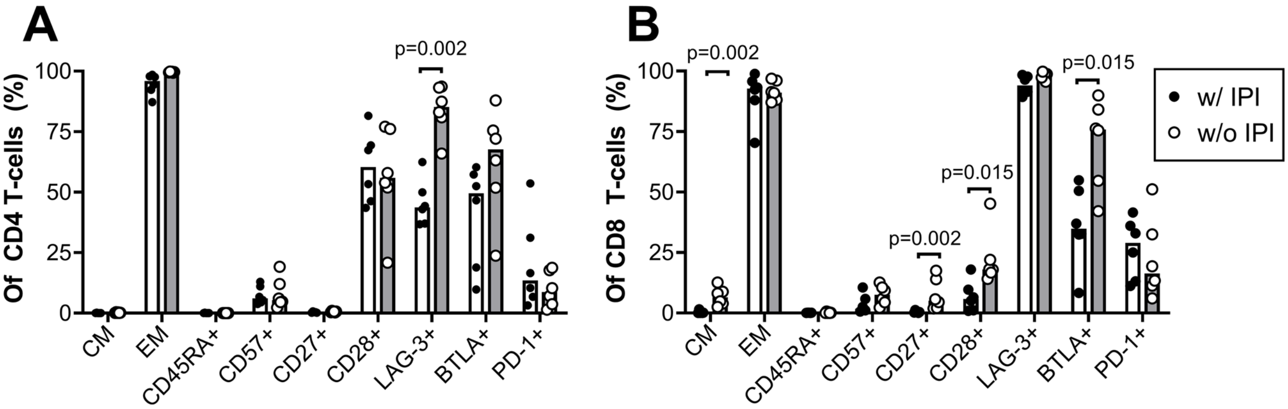

Supplementary Figure 3: Phenotype of expanded TILs with and without ipilimumab prior to tumor resection. Differences in CD4 (**A**) and CD8 (**B**) T cells in *ex vivo* expanded tumor infiltrating lymphocytes (REP-TILs) between patients with or without treatment with one dose ipilimumab prior to tumor resection. Mann–Whitney *U* test was used to test for significance. CM: Central memory (CD45RA-CCR7+); EM: Effector memory (CD45RA-CCR7-); IPI: Ipilimumab.

Supplementary Table 1: Antibody panels used in the flow cytometry analysis

Panel	Antibody	Flourochrome	Manufactor	Clone
1	CD45RA	FITC	BD	L48
2	CD57	FITC	BD	NK-1
3	CD16	FITC	Dako	DJ130c
4	LAG-3	FITC	LifeSpan	17B4
1	CD45RO	PE	BD	UCHL-1
2	CD27	PE	BD	L128
3	CD137	PE	BD	4B4-1
4	BTLA	PE	BD	J166-540
1, 2, 3, 4	CD4	PerCP	BD	SK3
1	CCR7	Pe-Cy7	BD	3D12
2	CD69	Pe-Cy7	BD	FN50
3	CD56	Pe-Cy7	BD	B159
4	PD-1	Pe-Cy7	BD	EH12.1
1	CD62L	APC	BD	DREG-56
2	CD28	APC	BD	CD28.2
3	TCRgd	APC	Biolegend	B1
4	TIM-3	APC	eBioscience	F38-2E2
1, 2, 3, 4	NIR (live-dead)	APC-Cy7	Invitrogen	n/a
1, 2, 3, 4	CD8	BV421	BD	RPA-T8
1, 2, 3, 4	CD3	BV510	BD	SK7

Supplementary Table 2: Antibody panel used in the mass cytometry analysis

Antibody	Metal conjugate	Manufactor	Clone
CD3	170-Er	Fludigm	UCHT1
CD4	145-Nd	Fludigm	RPA-T4
CD7	153-Eu	Fluidigm	CD7-6B7
CD8a	146-Nd	Fludigm	RPA-T8
CD8b	166-Er	eBioscience	SIDI8BEE
CD11b	144-Nd	Fluidigm	ICRF44
CD11c	162-Dy	Fluidigm	Bu15
CD14	160-Gd	Fluidigm	M5E2
CD16	148-Nd	Fluidigm	3G8
CD20	163-Dy	Biolegend	2H7
CD25	149-Sm	Fludigm	2A3
CD27	167-Er	Fludigm	O323
CD28	171-Yb	Biolegend	CD28,2
CD38	172-Yb	Fluidigm	HIT2
CD45	89-Y	Fludigm	HI30
CD45RA	169-Tm	Fluidigm	HI100
CD45RO	173-Yb	Biolegend	UCHL1
CD56	176-Yb	Fluidigm	NCAM16,2
CD103	155-Gd	Biolegend	Ber-ACT8
CD122	158-Gd	Biolegend	TU27
CD123	151-Eu	Fluidigm	6H6

CD127	165-Ho	Fluidigm	AO19D5
CD161	164-Dy	Fluidgim	HP-3G10
CD163	154-Sm	Fluidigm	GHI/61
CCR6	141-Pr	Fludigm	G034E3
CCR7	159-Tb	Fluidigm	G043H7
c-KIT	143-Nd	Fluidigm	104D2
CRTH2	156-Gd	Biolegend	BM16
HLA-DR	168-Er	Biolegend	L243
IgM	150-Nd	Biolegend	MHM88
KLRG-1	151-Dy	Miltenyi Biotech	REA261
NKG2a	142-Nd	Beckman Coulter	Z199
NKp44	147-Sm	Biolegend	P44-8
NKp46	174-Yb	Biolegend	9,00E+02
PD-1	175-Lu	Fluidigm	EH 12,2H7
TCR-gd	152-Sm	Fluidigm	11F2

Supplementary Table 3: Mass cytometry clusters of the *ex vivo* expanded tumor infiltrating lymphocytes (REP-TILs)

Cluster	Label	Expression of lineage markers	Median of CD3 (range)		
			**w/o ipilimumab**	**w/ ipilimumab**	**All**
1	CD4-EM	CD4, HLA-DR,	40.1% (14.2–75.2%)	35% (6.3–82.5%)	37.1% (6.3–82.5%)
2	CD4-CM	CD4, CCR7, HLA-DR	7.4% (2.8–9.9%)	6% (0.9–9.9%)	7.4% (0.9–9.9%)
3	CD4-NKT-like	CD4, CD56, CD45RA, CCR7, HLA-DR	2% (0.6–18.3%)	2.6% (1.6–6.8%)	2.5% (0.6–18.3%)
4	CD4-TCRgd	CD4, CD45RA, HLA-DR, TCRgd	0.4% (0.1–9.1%)	0.1% (0.1–4.0%)	0.3% (0.1–9.1%)
5	CD8-EM	CD8, HLA-DR	21.6% (8.2–62.3%)	31.8% (3.9–57.7%)	29.2% (3.9–62.3%)
6	CD8-CM	CD8, CCR7, HLA-DR	2.2% (1.0–13.1%)	3.2% (0.4–19.6%)	3% (0.4–19.6%)
7	CD8-NKT-like	CD8, CD56, HLA-DR	7.0% (0.7–17.5%)	11.4% (1.6–35.5%)	8% (0.7–35.5%)
8	CD8-TCRgd	CD8, HLA-DR, TCRgd	0.5% (0–3.7%)	0.5% (0.2–3.2%)	0.5% (0.0–3.7%)

Description and size of the 8 primary clusters from the multidimensional semi-supervised cluster analysis of the REP-TILs. CM: Central memory (CD45RA-CCR7+); EM: Effector memory (CD45RA-CCR7-).
